# Mental Nursing

**Published:** 1898-01-08

**Authors:** 


### EDITOR'S LETTER-BOX.

[Our Correspondents are reminded that prolixity is a great bar to publication, and that brevity of style and conciseness of statement greatly facilitate early insertion.]

### MENTAL NURSING.

A MALE ATTENDANT writes:—When a man enters upon the duties of an attendant on the insane he is little conscious of the responsibilities and difficulties he has at all periods to contend with. Firstly, he has to endure the insults, violence, and everything that tends to make an unpleasant feeling, and never is it his duty to retaliate in like manner. We, of course, know full well that these unfortunate creatures are not fully responsible for their conduct, and further, we know that we are, as attendants, caring for those who through affliction are totally unable to take care of themselves. The attendant is at all times to be an example of good temper, forbearance, and kindness; and, indeed, that is far less than what is expected of him; he has to hold himself responsible for anything that may tend to injure the patient, he is expected to answer correctly for any accident that may occur, fractures, and marks of all descriptions which so often occur in general paralytics, epileptics, and all feeble patients of all mental diseases. He has at all times to attend in precisely the same form as a mother would to her child, and to perform his duties to the entire satisfaction of his superior officer. To master these duties in a loyal and upright manner, and to thoroughly understand the ways of his patients and the various routine of duty that he is daily called upon to attend, he, like others in another branch of business, has to be taught and trained. But when he has served his institution, we will say from two to three years, he during that period has, through his study and perseverance, succeeded in gaining the certificate of proficiency in mental nursing; he may then take upon himself the responsible position of nursing in private houses. So I wish to show briefly the difference in nursing in an asylum and that of a private house. In an asylum the attendant is called to his duty at 6.30 a.m. If he be on duty in the imbecility wards he has to wash, dress, and make, we will say, twenty patients neat, clean, and comfortable by 7.30 for breakfast; at 8.30 it is his duty to see that each patient in his day-room is ready for medical inspection at 10 a.m.; he then is required to have his galleries, day-rooms, dormitories, in a sweet and neat condition by noon; at 1 p.m. dinner is served to patients, taking care that each patient partakes of his food in a quiet manner. So much, then, for the morning's routine. When duties are less difficult, his duty in the afternoon is merely to watch his patients at exercise in the airing courts provided for the purpose, to guard against escapes, quarrels, and accidents. Tea-time arrives at 5.30, bed at 7.30, when each patient is carefully examined to trace any marks, &c.; each patient's suit of clothes are carefully searched, and when this is done the attendant is at liberty until 10 p m. So much, then, for the asylum duties. In private nursing, duty is mostly of a different nature, inasmuch that the patient himself is usually harder to control and more apt to resist interference with his liberty. The relatives of the patient will often be suspicious or be fussy, or positively obstrusive; the labour is more exhausting, being often night and day work; the risks are far greater, from stairs, knives, &c., and when the patient becomes excited and at times violent he has only himself to guard against these attacks; and should an accident occur at night he must himself use first aid until the arrival of a doctor. So I advise all intending attendants that cannot refrain from drink never to undertake these very responsible duties. They will find their duties are of less trouble should they keep from alcoholic drinks. I consider that a man ought not to enter a private house that cannot do without these drinks. His position is one of great trust and responsibility, and he should at all times consider himself the right hand friend of his patient, and in the end not only will he feel in himself a pleasure that he has done his duty to his fellow creature, but will be a credit to the institution to which he may belong. In conclusion, I think that when parents and friends of the patient leave the patient entirely to the mercy and care of his attendant, that they themselves will in the end derive and yield a beneficial result; and, lastly, I consider that a good attendant is worthy of all encouragement in private houses.

